# Morphine Withdrawal Enhances HIV Infection of Macrophages

**DOI:** 10.3389/fimmu.2019.02601

**Published:** 2019-11-15

**Authors:** Xu Wang, Jinbiao Liu, Lina Zhou, Wen-Zhe Ho

**Affiliations:** Department of Pathology and Laboratory Medicine, Lewis Katz School of Medicine, Temple University, Philadelphia, PA, United States

**Keywords:** morphine, abrupt withdrawal, precipitated withdrawal, microRNAs, HIV, macrophages

## Abstract

Opioid withdrawal recurs at high rates in opioid use disorder and compromises the immune system. In general, there are two types of opioid withdrawal: abrupt withdrawal (AW) and precipitated withdrawal (PW). In this study, we examined the effect of morphine AW or morphine PW on HIV infection of human blood monocyte-derived macrophages. We observed that both morphine AW and PW enhanced the susceptibility of macrophages to HIV infection. In addition, both AW and PW activated HIV replication in the latently infected myeloid cells (U1 and OM10.1). Investigation of mechanisms responsible for these observations showed that both AW and PW could inhibit the expression of multiple intracellular HIV inhibitory factors, including APOBE3G/F, SAMHD1, MX2, and HIV restriction microRNAs (miR-28, miR-125b, and miR-150) in macrophages. These findings provide additional evidence to support the notion that opioid use compromises the intracellular anti-HIV immunity and facilitates HIV infection and persistence in macrophages.

## Introduction

Opioid withdrawal recurs at high rates in opioid use disorder and compromises the immune system (1, 2). Opioid tolerance has been studied extensively; once tolerance develops, discontinuing the drug with or without administering an opioid antagonist can progress to a state of physical dependence (2). While there are very few studies that examine the impact of morphine withdrawal on host immunity and HIV infection, it is known that opiate addiction compromises the immune system and facilitates HIV infection and replication. Early clinical and epidemiologic studies show that opioid use is a cofactor in the pathogenesis of HIV disease. Opioids modulate cytokine production/cell trafficking and, thereby, increase the vulnerability of the host cells to HIV infection (3, 4). Our earlier studies documented that morphine or methadone enhances HIV infection of macrophages and microglia (5–7). We also showed that heroin use facilities HIV infection of macrophages through inhibiting the expression of the HIV restriction miRNAs (8). These findings are clinically relevant and significant as macrophages have an important role in HIV infection during all stages of the disease where they act as key target cells and reservoirs, a means to other tissues in the body, and viral transmitters to CD4^+^ T cells.

The enhancing effect of opiates on HIV infection/replication is likely due to their negative impact on host immunity (9–11). The impact of opiates on the immune system has been extensively studied in T cells, B cells, macrophages, natural killer cells, and polymorphonuclear leukocytes (12–15). Although studies have demonstrated that opioids modulate the functions of macrophages, there is limited information about specific mechanism(s) of opioid effect on the intracellular immunity against HIV in macrophages. Previous studies concluded that morphine inhibited the production of type I interferons (IFNs) (16), the primary cytokines that regulate all immune stages and lead host innate immunity against viral infections. Upon exposure to viral infection, IFN-α/β can activate downstream cell signaling, inducing multiple IFN-stimulated genes and other antiviral factors, including those HIV restriction miRNAs. Thus, it is of significance to examine whether opioid withdrawal inhibits intracellular anti-HIV immunity in macrophages and facilitates HIV infection and replication.

## Materials and Methods

### Primary Cells and Cell Lines

Purified primary human monocytes from peripheral blood were obtained from the Human Immunology Core at the University of Pennsylvania (Philadelphia, PA, USA). The Human Immunology Core has the Institutional Review Board approval for blood collection from healthy donors. Freshly isolated monocytes were plated in 48-well culture plates (Corning CELLBIND Surface) at a density of 2.5 × 10^5^ cells/well in DMEM containing 10% fetal calf serum (FCS), 1% non-essential amino acid, 1% L-glutamine, and 1% penicillin–streptomycin solution at 37°C with 5% CO_2_. Monocyte-derived macrophages refer to 7-day-cultured monocytes *in vitro*. HIV-infected U1 and OM10.1 cell lines were provided by the AIDS Reagent Program, National Institutes of Health. U1 is a cloned cell line derived by limiting dilution cloning of U937 cells surviving an acute infection with HIV (LAV-1 strain); each cell has two copies of integrated HIV proviral DNA (17, 18). OM10.1 cells were cloned from HL-60 promyelocyte cells that endured an acute HIV infection. Each cell contains a single integrated provirus (19, 20). U1 and OM10.1 cells were cultured in RPMI1640 supplemented with 10% FCS, 1% L-glutamine, and 1% penicillin–streptomycin.

### Reagents and HIV Strain

Morphine sulfate was provided by the National Institute of Drug Abuse (Rockville, MD). Naloxone was obtained from Sigma (St. Louis, MO). The macrophage-tropic R5 strain (Bal), an isolate from human infant lung tissue, was obtained from the AIDS Reagent Program, the National Institutes of Health, Bethesda, MD.

### Morphine Treatment and Withdrawal

**Figure 1:**
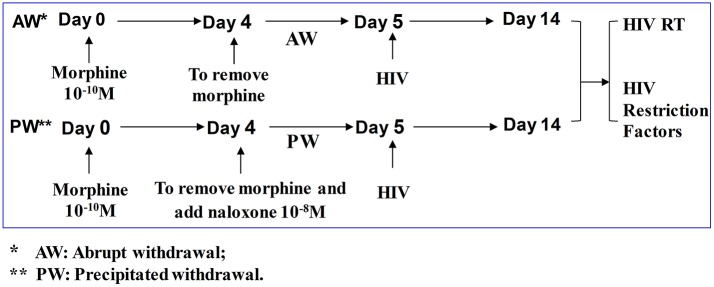
Experimental design to determine the effect of morphine withdrawal on HIV infection of primary human macrophages. Seven-day-cultured macrophages were incubated with or without morphine (10^−10^ M) for 96 h and then subjected to morphine AW or PW for 24 h. For AW, we removed morphine from the cell cultures by washing the cells three times with plain DMEM. For PW, the cells were treated with naloxone (10^−8^ M) 3 min after morphine AW. The cells undergoing morphine AW or PW as described above were infected with equal amounts (HIV p24, 20 ng/10^6^ cells) of cell-free HIV Bal strain for 2 h. The cells were then washed three times with plain DMEM to remove unabsorbed HIV 24 h after infection. Supernatants were collected from HIV-infected cell cultures for HIV RT activity assay at day 9 post-infection (day 14 after morphine treatment).

Seven-day-cultured macrophages (2.5 × 10^5^ cells/well in 48-well plates) were treated with or without morphine (10^−10^ M) for 96 h and morphine was then removed. For morphine abrupt withdrawal (AW), we removed morphine from the cell cultures by rigorously washing the cells three times with plain DMEM. For precipitated withdrawal (PW), morphine was removed from the cultures and then cells were treated with naloxone (10^−8^ M) (Figure 1). Cells were then subjected to RNA extraction and real-time RT-PCR at the time points indicated in the figure legends. U1 and OM10.1 cells in T-25 flasks were incubated with or without morphine (10^−8^ M) for 96 h and then plated in triplicate at a density of 10^5^ cells/well in 48-well culture plates. Afterward, they were subjected to either AW or PW. The cells were stimulated with TNF-α (2 ng/ml) 2 h post morphine withdrawal. HIV RT activity in the culture supernatant was measured at 48 h post morphine withdrawal.

### HIV Infection

Seven-day-cultured macrophages (2.5 × 10^5^ cells/well in 48-well plates) were treated with or without morphine (10^−10^ M) for 96 h and then morphine was removed from the cell cultures for 24 h. The cells were infected with equal HIV Bal strain (p24 protein, 20 ng/10^6^ cells) for 2 h at 37°C. After infection, the cells were then washed three times with plain DMEM to remove the unabsorbed viruses. The final wash was tested for viral RT activity and found to be free of residual inoculum, as compared with untreated cells. Culture supernatant was collected at day 9 post-infection for HIV RT activity assay.

### HIV Reverse Transcriptase (RT) Assay

Determination of HIV RT activity was adapted from Willey et al. (21). In brief, collected culture supernatant (10 μl) was added to a cocktail containing poly (A) (GE Healthcare, Mickleton, NJ), oligo (dT) (USB, Cleveland, OH), MgCl_2_, and ^32^P dTTP (PerkinElmer, Boston, MA) and incubated for 20 h at 37°C. The cocktail (30 μl) was spotted onto DE81 paper, dried, and washed five times with 2 × saline-sodium citrate buffer and once with 95% ethanol. The filter paper was then air-dried. Radioactivity was counted in a liquid scintillation counter (PerkinElmer, Boston, PA).

### RNA Extraction and Reverse Transcription

Total cellular RNA was isolated from macrophages using Tri-reagent (Molecular Research Center, Cincinnati, OH). In brief, the total cellular RNA was extracted by a single step, guanidium thiocyanate-phenol-chloroform extraction. After centrifugation at 13,000 g for 15 min at 4°C, the RNA-containing aqueous phase was collected and precipitated in isopropanol. The RNA precipitates were then washed once in 75% ethanol and re-suspended in 30 μl of RNase-free water. Total RNA (1 μg) was subjected to reverse transcription using the reverse transcription system (Promega, Madison, WI) according to the manufacturer's protocol. The cDNA was ready to serve as a template for PCR amplification. For miRNA reverse transcription, the total RNAs, including microRNAs, were reversely transcribed with miScript Reverse Transcription Kit (QIAGEN, Germantown, MD, USA).

### qRT-PCR for mRNA and microRNA

Real-time PCR was performed with the iQ SYBRGreen Supermix (Bio-Rad Laboratories, Hercules, CA, USA) as previously described. The oligonucleotide primers were synthesized by Integrated DNA Technologies, Inc. (Coralville, IA, USA), and sequences will be available upon request. Real-time PCR for the quantification of microRNAs (miRNA-28, miRNA-125b, miRNA-150, miRNA-132, miRNA-124a, Let-7C, and RNU5A) was conducted with miScript Primer Assays and miScript SYBR Green PCR Kit (QIAGEN, Germantown, MD, USA) as instructed by the manufacturer.

### Statistical Analysis

Where applicable, data were expressed as mean ± SD. To compare the mean of the two groups (AW or PW treated vs. untreated control cells), statistical significance was measured by ANOVA with the appropriate *post-hoc* test. Statistical analyses were conducted with SPSS 18.0 software (SPSS Inc., Chicago, IL, USA). Statistical significance was defined as *p* < 0.05.

## Results

### Effect of Morphine AW or PW on Acute and Latent HIV Infection

**Figure 2:**
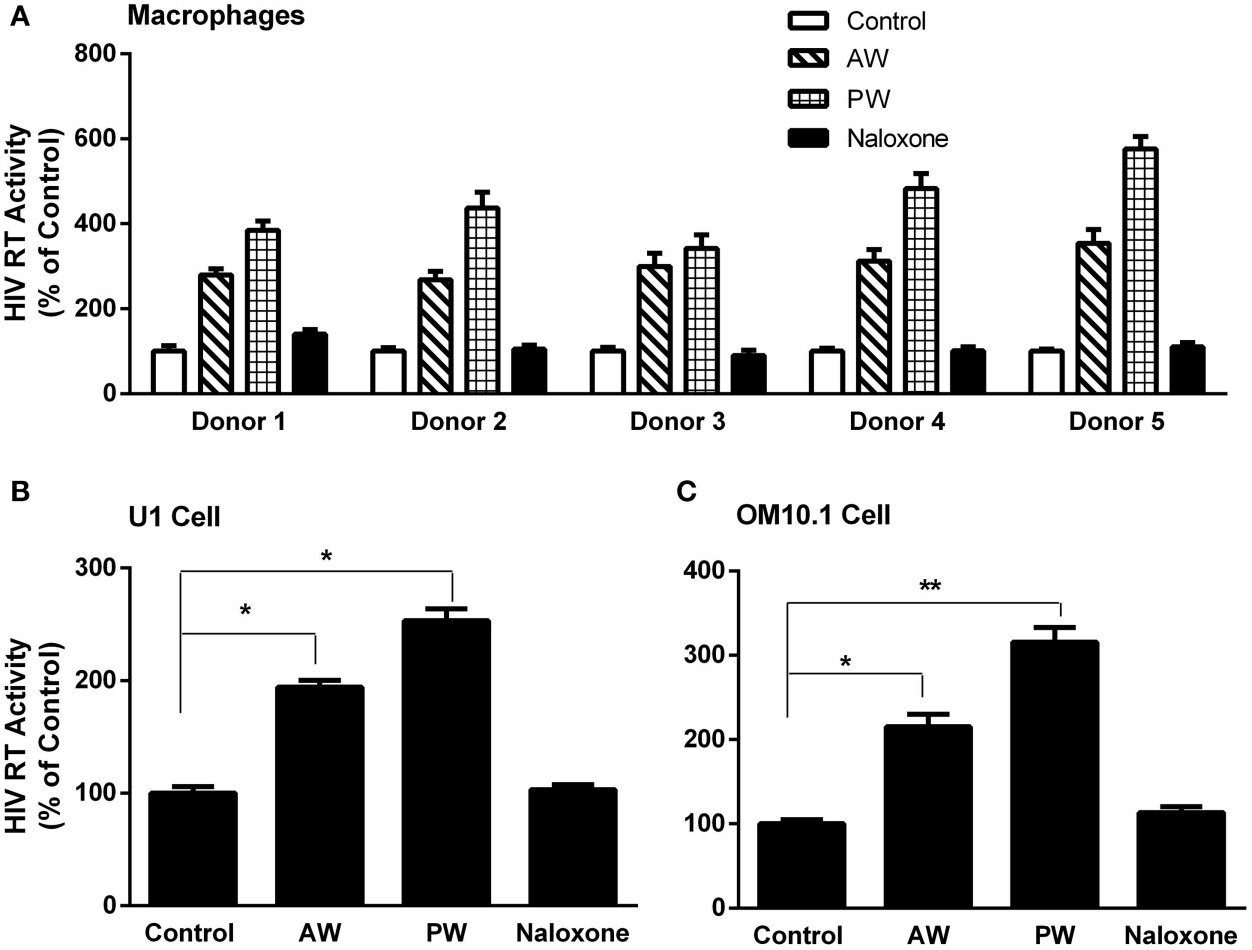
Effect of AW or PW on HIV replication in macrophages, U1, and OM10.1 cells. **(A)** Seven-day-cultured macrophages were incubated with or without morphine (10^−10^ M) for 96 h, followed by morphine AW or PW for 24 h. For PW, naloxone (10^−8^ M) was added to the cell cultures 3 min after morphine was removed. The cells were then infected with HIV (Bal) for 2 h. HIV RT activity was measured in culture supernatant collected at day 9 after infection. The data shown are the mean ± SD of triplicate determinations, five independent experiments using macrophages from five different donors. **(B,C)** U1 cells and OM10.1 cells were incubated with or without morphine (10^−8^ M) for 96 h and then subjected to morphine AW or PW. The cells were stimulated with TNF-α (2 ng/ml) 2 h post-withdrawal. HIV RT activity was measured in culture supernatant 48 h post-withdrawal. The data shown are the mean ± SD of triplicate determinations, representative of three independent experiments (^*^p < 0.05, ^**^p < 0.01, AW or PW vs. control).

We first examined whether AW or PW enhances acute HIV infection of primary human macrophages. As demonstrated in Figure 2A, either AW or PW could enhance the HIV infection of macrophages. We also examined the effect of morphine AW or PW on TNF-α-induced HIV replication in latently infected cell lines. As shown in Figures 2B,C, both morphine AW and PW could activate and enhance TNF-α-induced HIV replication in U1 and OM10.1 cells.

### Effect of Morphine AW or PW on miRNAs Related to HIV Infection in Macrophages

**Figure 3:**
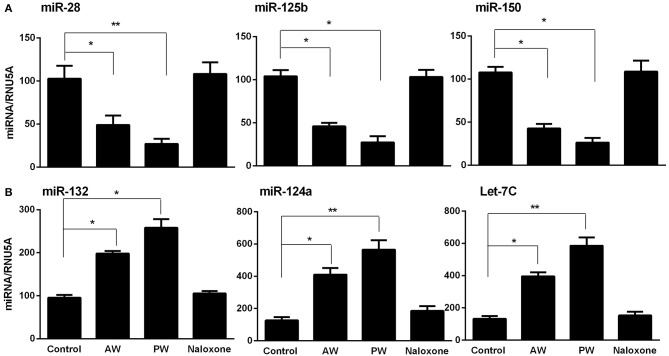
Effect of AW or PW on HIV restriction miRNAs and HIV replication-required miRNAs in macrophages. Seven-day-cultured macrophages were incubated with or without morphine (10^−10^ M) for 96 h, followed by morphine AW or PW for 6 h. For PW, naloxone (10^−8^ M) was added to the cell cultures 3 min after morphine was removed. Total cellular RNA extracted from cell cultures and subjected to real-time RT-PCR for miRNA expression, **(A)** miR-28, miR-125b, and miR-150, **(B)** miR-132, miR-124a, and Let-7C. The data shown are the mean ± SD of triplicate cultures, representative of three experiments using cells from three different donors (^*^p < 0.05, ^**^p < 0.01, AW or PW vs. control).

Our early study (22) demonstrated that freshly isolated monocytes from human blood expressed significantly higher levels of the cellular anti-HIV miRNAs (miRNA-28, miRNA-150, miRNA-223, and miRNA-382) than donor matched macrophages. These miRNAs play a key role in suppressing HIV replication in monocytes and macrophages (11, 22). Thus, we examined whether morphine AW or PW inhibits the expression of these HIV restriction mRNAs in macrophages. As shown in Figure 3, both morphine AW and PW significantly decreased the expression of the HIV restriction miRNA (miR-28, miR-125b, and miR-150) (Figure 3A) and increased HIV enhancing miRNA (23, 24) (miR132, miR124a, and let-7C) in macrophages (Figure 3B).

### Effect of Morphine AW or PW on HIV Restriction Factor in Macrophages

**Figure 4:**
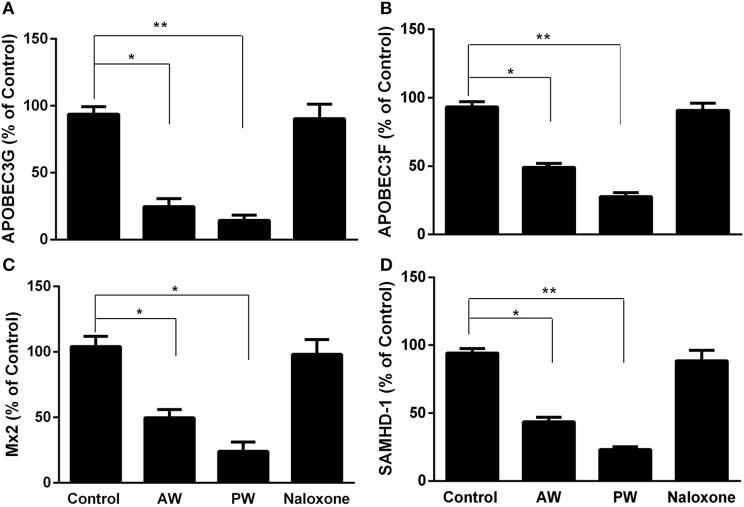
Effect of AW or PW on cellular anti-HIV factor expression in macrophages. Seven-day-cultured macrophages were incubated with or without morphine (10^−10^ M) for 96 h, followed by AW or PW for 6 h. For PW, naloxone (10^−8^ M) was added to the cell cultures 3 min after morphine was removed. Total cellular RNAs were then extracted from cell cultures 6 h post-withdrawal and subjected to real-time RT-PCR for the mRNA expression of APOBEC3G **(A)**, APOBEC3F **(B)**, SAMHD-1 **(C)**, and Mx2 **(D)**. The data shown are the mean ±SD of triplicate cultures, representative of three experiments using cells from three different donors (^*^p < 0.05, ^**^p < 0.01, AW or PW vs. control).

We next examined the effect of morphine AW or PW on the expression of the intracellular HIV restriction factors. As shown in Figure 4, both morphine AW and PW significantly inhibited the expression of apolipoprotein B mRNA-editing enzyme, catalytic polypeptide-like 3G/F (APOBEC3G/F), SAM domain and HD domain-containing protein 1 (SAMHD1), and MX dynamin-like GTPase 2 (MX2) at 6 h post morphine withdrawal.

## Discussion

In this study, we demonstrated that both morphine AW and PW significantly enhanced acute HIV infection of primary human macrophages (Figure 2A). In addition, we found that either morphine AW or PW could activate HIV replication in latently infected myeloid cells (U1 and OM10.1, Figures 2B,C). These observations support an early *in vivo* study using the non-human primate model, showing that precipitated morphine withdrawal (administration of an opioid antagonist with or without discontinuing the morphine) increases viral load in SIV-infected monkeys (25). We previously showed that morphine withdrawal manifests the enhancing effect on HIV infection of human T lymphocytes (26). Mechanistically, we observed that both morphine AW and morphine PW significantly inhibited the expression of the HIV restriction miRNAs (Figure 3A) and increased the expression of HIV replication-required miRNAs (Figure 3B). Studies have demonstrated that many miRNAs participate in the host immune responses to HIV infection (22, 27). In general, the miRNAs that interfere with HIV replication can bind to viral RNAs or indirectly disrupt viral progression by targeting the cellular factors associated with HIV replication (28–30). Studies have identified multiple cellular miRNAs (miR-28, 29a, 125b, 150, 198, 223, and 382) to target a set of accessory genes of HIV (31–34). For example, HIV restriction miRNAs target the 3′UTR of HIV transcripts converting productive HIV replication into latency stage in resting CD4^+^ T cells (27). We showed that some of these miRNAs contribute to the resistance of monocytes to HIV infection (22). In addition to the inhibitory effect on the HIV restriction miRNAs, both morphine AW and PW could suppress the expression of HIV restriction cellular factors (APOBEC3G/F, SAMHD1, and MX2) (Figure 4). APOBEC3G and APOBEC3F, the key members of the APOBEC3 family, have the ability to inhibit HIV mobility (35–37). It is known that SAMHD1 can reduce the dNTPs pool in macrophages by hydrolyzing dNTPs into their precursors (nucleosides and triphosphates), resulting in inefficient HIV reverse transcription (38, 39). Mx2 is a HIV post-entry inhibitor that blocks the capsid-dependent nuclear import of subviral complexes (40–42). Given the significant impact of these intracellular antiviral factors in the control of HIV replication, it is likely that the suppression of these elements in the HIV target cells is a sound mechanism for morphine AW- or PW-mediated enhancing effect on HIV replication in macrophages and the latently infected cells. Although these *in vitro* systems cannot reflect the *in vivo* situation where opioid withdrawal occurs repetitively during the long course of opioid abuse, they provide direct and initial evidence about the effect of morphine withdrawal on the intracellular immunity and HIV replication in macrophages, a key target and reservoir of HIV.

In summary, these data in conjunction with our earlier reports (8, 43) clearly demonstrate that opioid withdrawal has a negative impact on host cell innate immunity against HIV, resulting in HIV infection and persistence in the primary target cells. While future *in vivo* studies are necessary in order to confirm our *in vitro* observations, these findings have provided additional experimental evidence to support the notion that opioid abuse not only contributes to HIV transmission but also facilitates HIV replication in the target cells.

## Data Availability Statement

The datasets generated for this study are available on request to the corresponding author.

## Author Contributions

XW, LZ, JL, and W-ZH conceived and designed the experiments. XW, LZ, and JL performed the experiments. XW and LZ analyzed the data. XW created the figures. W-ZH contributed reagents, materials, and analysis tools. XW and W-ZH wrote the manuscript. All authors reviewed the manuscript.

### Conflict of Interest

The authors declare that the research was conducted in the absence of any commercial or financial relationships that could be construed as a potential conflict of interest.
